# Nervonic acid reduces the cognitive and neurological disturbances induced by combined doses of D‐galactose/AlCl_3_ in mice

**DOI:** 10.1002/fsn3.3533

**Published:** 2023-07-06

**Authors:** Mayile Aihaiti, Haidan Shi, Yaojie Liu, Chen Hou, Xiaoyu Song, Mengting Li, Jianke Li

**Affiliations:** ^1^ College of Food Engineering and Nutritional Science Shaanxi Normal University Xi'an China; ^2^ University Key Laboratory of Food Processing Byproducts for Advanced Development and High Value Utilization, Shaanxi Normal University Xi'an China

**Keywords:** cognitive, hippocampus, nervonic acid, neurotransmitter, oxidative stress

## Abstract

Nervonic acid (NA) is a kind of ultra‐long‐chain monounsaturated fatty acid, which can repair nerve cell damage caused by oxidative stress. Alzheimer's disease (AD) is a nervous system disease and often accompanied by the decline of learning and memory capacity. In this study, the combined dose of D‐galactose/AlCl_3_ was used to establish a mouse model of AD. Meanwhile, the mice were treated with different doses of NA (10.95 and 43.93 mg/kg). The results showed that NA delayed the decline of locomotion and learning ability caused by D‐galactose/AlCl_3_, increased the activity of total superoxide dismutase, catalase, glutathione peroxidase, and reduced the content of malondialdehyde in vivo. Besides, NA reduced the levels of interleukin‐6 (IL‐6), tumor necrosis factor‐α (TNF‐α), interleukin‐1β (IL‐1β), aspartate aminotransferase, alanine aminotransferase, increased the levels of 5‐hydroxytryptamine, dopamine, γ‐aminobutyric acid, alleviated the cell morphology damage induced by D‐galactose/AlCl_3_ in hippocampus and liver tissue. Furthermore, the intervention of NA upregulated the expression levels of PI3K, AKT, and mTOR genes and downregulated the expression levels of TNF‐α, IL‐6, and IL‐1β genes. Therefore, we speculate the intervention of NA could be an effective way in improving cognitive impairment through the activation of PI3K signaling pathway. These results suggest that NA has the potential to be developed as antioxidant drug for the prevention and early therapy of AD.

## Graphical abstract

Nervonic acid (NA) is a kind of ultra‐long‐chain monounsaturated fatty acid which can repair nerve cell damage caused by oxidative stress. In this study, the combined dose of D‐galactose/AlCl_3_ was used to establish a mouse model of Alzheimer's disease. These results suggest that NA has the potential to be developed as antioxidant drug for the prevention and early therapy of AD.

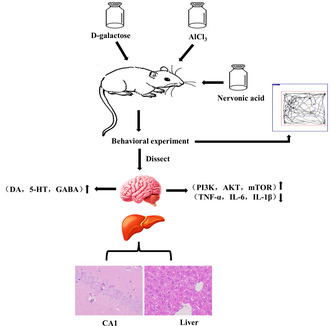

## 1 INTRODUCTION

AD is an irreversible progressive neurological disease. According to the data from Alzheimer's disease (AD) international, about 50 million people worldwide are suffered from it (Guo et al., 2020; Van Dyke et al., 2021; Winstone et al., 2022). Meanwhile, it is characterized by the damage of hippocampus neuron cells, such as the occurrence of irregular cell phenomenon (Antonovaite et al., 2021; Iwakiri et al., 2009). In addition, a study finds that the decline of neurotransmitter levels can accelerate the onset of AD, including DA, 5‐hydroxytryptamine (5‐HT), and γ‐aminobutyric acid (GABA) (Manzoor & Hoda, 2020). Besides, the excessive production of reactive oxygen species (ROS) will destroy the normal morphology of nerve cells and cause excessive activation of the immune system in vivo (Liu, Han, et al., 2020; Liu, Wang, et al., 2020). Notably, D‐galactose can accelerate the overproduction of ROS, and AlCl_3_ intervention can cause neurotoxicity in mice (Xing et al., 2018; Zhong et al., 2020). Therefore, the evidence shows that D‐galactose/AlCl_3_ combined treatment can induce AD‐like symptoms, including cognitive and memory impairments, oxidative damage, and inflammation (He et al., 2022; Liu et al., 2022; Song et al., 2022; Zhang et al., 2020).

Nervonic acid (NA) is a kind of ultra‐long‐chain monounsaturated fatty acid (Umemoto et al., 2021). It was first isolated in the brain tissue and has a variety of biological activities (Li et al., 2019; Liu, Han, et al., 2020; Liu, Sun, et al., 2021; Liu, Wang, et al., 2020; Liu, Wang, et al., 2021). For example, NA alleviated the adverse effects of senile dementia on motor ability and improved the capacity of autonomous exploration in mice (Hu, Cui, et al., 2021, Hu, Wharton, et al., 2021). Simultaneously, it was found that the NA intervention enhanced the activity of SOD and catalase (CAT) in experimental autoimmunity encephalomyelitis mice (Liu, Sun, et al., 2021; Liu, Wang, et al., 2021). Moreover, the intervention of NA increased DA and 5‐HT levels in 1‐Methyl‐4‐phenyl‐1,2,3,6‐tetrahydropyridine induced C57BL/6 mice (Hu, Cui, et al., 2021; Hu, Wharton, et al., 2021). Finally, NA (197 mg/kg) intervention reduced the level of TNF‐α in serum, improved the abnormal cell morphology caused by myelin oligodendrocyte glycoprotein peptides 35–55, and no side effects were found (Liu, Sun, et al., 2021, Liu, Wang, et al., 2021).

In previous reports, the activation of PI3K pathway can promote the production of antioxidant enzymes in vivo, such as SOD, glutathione peroxidase (GSH‐PX), and CAT (Li et al., 2022; Lu et al., 2021; Sugimoto et al., 2021). Meanwhile, the study finds that the activation of PI3K pathway can alleviate morphological damage in hippocampal cells caused by streptozotocin intervention in C57BL/6 mice (Li et al., 2021).

In this study, the motor and learning abilities of experimental mice were evaluated through open‐field and Barnes maze tests. In addition, some biochemical experiments and hematoxylin–eosin staining (HE) were conducted to explore the antioxidant and anti‐inflammatory capabilities of NA. Finally, RT‐qPCR was conducted in order to further verify our experimental conjecture.

## 2 MATERIALS AND METHODS

### 2.1 Materials

NA (purity ≥ 99%), D‐galactose (purity ≥ 99%), and AlCl_3_ (purity ≥ 99%) were purchased from Sigma‐Aldrich. The assay kits for total superoxide dismutase (T‐SOD) (A001‐1‐2), GSH‐PX (A005‐1‐2), CAT (A007‐1‐1), malondialdehyde (MDA) (A003‐1‐2), alanine aminotransferase (ALT) (C009‐2‐1), and aspartate aminotransferase (AST) (C010‐2‐1) were obtained from Nanjing Jiancheng Bioengineering Institute. The TNF‐α, IL‐1β, interleukin‐6 (IL‐6), 5‐HT, DA, and GABA ELISA assay kits were purchased from Shanghai Enzyme‐linked Biotechnology. These kits of TRIzol extraction, Reverse Transcription (HiscriptIIQ RT Super Mix), and Quantitative PCR (Cham QTM SYBR qPCR Master Mix) were purchased from Vazyme.

### 2.2 Animals and treatment

Male C57BL/6 mice (7 weeks old) were obtained from the Central Animal House Facility of Shaanxi Normal University, Xian, China (Approval Number: SYXK2021‐003). The mice were fed in the Animal Breeding Center of Shaanxi Normal University (temperature was 25 ± 2°C, humidity was 55%–65%, 12 h light/dark cycle) and were provided with adequate food and water. All animal procedures complied with the Guide for the Care and Use of Laboratory Animals and were approved by the Animal Care and Use Committee of Shaanxi Normal University.

After 5 days of adaptation, animals were randomly divided into four experimental groups (*n* = 12 in each group), which were named as NC (normal control group), MOD (AD model group), LN (low‐dose NA intervention group), and HN (high‐dose NA intervention group), respectively. Meanwhile, D‐galactose and AlCl_3_ were dissolved in normal saline. Among them, mice of NC group were given normal saline by gavage, while other mice were treated with D‐galactose (120 mg/kg) and AlCl_3_ (20 mg/kg) for 8 weeks. According to the references, cognitive impairment and AD pathology have been established in this model (Liu et al., 2022; Song et al., 2022; Zhang et al., 2020). From the fifth week to the eighth week, the mice of MOD group were gavaged with normal saline, and the mice of LN and HN groups were treated with NA for 10.95 and 43.93 mg/kg, respectively. The dose of NA was identified according to the previous study (Hurtado et al., 2015; Wu et al., 2020). In addition, initial and final weights of mice were recorded.

### 2.3 Open‐field test (OFT)

OFT is a method to evaluate the autonomous behavior of experimental animals in a strange environment (Knight et al., 2021; Wang et al., 2021). Briefly, a carton was prepared (length, width, and height were 30 cm), and a square area (both length and width were 15 cm) was determined in the center of the bottom. Meanwhile, the mice were placed in the center of the bottom and allowed to freely move for 10 min. During the experiment, the defecation quantity, time spent in the central area, total distance, and movement track of the mice were recorded by Smart video tracking system v 3.0 (Panlab). In addition, 75% alcohol was sprinkled after each test, which can exclude the interference of odor on mice behavior.

### 2.4 Barnes maze test (BM)

BM is a method to evaluate the cognitive and memory ability of mice (Pitts, 2018; Russo‐Savage et al., 2020). In this study, the instrument was installed on a rotatable wooden support system (the height of Barnes maze was 99 cm and diameter 122 cm). Meanwhile, all mice were adapted for 1 h before each experiment. During the period of training, the mice were placed in the center of the instrument, and the experiment ended when the mice went into the escape tunnel. If the mice do not find the escape tunnel within the specified time, they will be placed in the escape tunnel for 30 s. On the fourth day of BM, these indices of total distance traveled, the number of errors, and time to escape tunnel were recorded by Smart 3.0 tracking system (Panlab). In addition, 75% alcohol was sprinkled after each test, which can exclude the interference of odor on mice behavior.

### 2.5 Determination of biochemical indicators

After behavioral tests, all mice were sacrificed after hypodermic injection of pentobarbital sodium (40 mg/kg). Meanwhile, the blood was obtained and supernatant was stored at −80°C after centrifugation (4°C, 3000 r/min, 10 min). In addition, some fresh brain and liver tissue were immediately removed for further biochemical analysis and stored at −80°C. Furthermore, the measurement procedures of T‐SOD, CAT, GSH‐PX, MDA, IL‐1β, IL‐6, TNF‐α, 5‐HT, DA, and GABA levels were conducted following the manufacturer's instructions. The liver and whole brain of mice were stored in 4% paraformaldehyde for histopathological examination. Finally, the thymus and liver of mice were weighed, and the organ index was calculated according to the following formula: Organ index (mg/g) = viscera weight (mg)/mouse body weight (g).

### 2.6 Hematoxylin–eosin staining (HE staining)

According to the previous research methods, the pathological histomorphology of liver and brain tissues was analyzed (Minamisawa et al., 2021). Briefly, after dehydration, dewaxing, embedding, sectioning, staining, and sealing, the liver and brain sections were obtained. The slides were examined under an optical microscope (Olymups) and cell morphology was analyzed by using ImageJ 1.44p software (National Institutes of Health).

### 2.7 Reverse transcription and quantitative real‐time PCR (RT‐qPCR)

The gene expression of β‐actin, IL‐1β, IL‐6, TNF‐α, PI3K, AKT, and mTOR was determined using quantitative real‐time PCR instrument (USA Biorad, CFX96). The forward and reverse primers for the tested genes are listed in Table 1. Briefly, total RNA of the brain tissue was extracted by using the kit of TRIzol extraction. After synthesizing cDNA, the transcription of all the genes was performed using a Maxima SYBR green/ROX qPCR master mix. The quantification of target mRNA expression was performed using the 2^−ΔΔCT^ method by β‐actin as reference and MOD group as control, which was standardized to 1.

**TABLE 1:** The information of primer sequence.

Primers	Forward	Reverse
β‐Actin	TACCACAGGCATTGTGATGG	TTTGATGTCACGCACGATTT
TNF‐α	CTCTTCTGCCTGCTGCACTTTG	ATGGGCTACAGGCTTGTCACTC
IL‐6	CCACGGCCTTCCCTACTTC	CTCATTTCCACGATTTCCCAG
IL‐1β	CCACAGACCTTCCAGGAGAATG	GTGCAGTTCAGTGATCGTACAGG
PI3K	TCCAAATACCAGCAGGATCA	ATGCTTCGATAGCCGTTCTT
mTOR	GCTCCTGGGTGAGAGAGCTG	CAGGCTGCTGGAGCTTGTTG
AKT	GAACGGCCTCAGGATGTGGA	GGTGCGCTCAATGACTGTGG

### 2.8 Statistical analysis

Statistical analysis and plots were performed using Graph Pad Prism 8.0. The results of the biochemical are expressed as mean ± SD. The statistical differences between the two groups were analyzed using one‐way ANOVA with Tukey's multiple comparisons and *p*‐value <.05 was considered statistically significant.

## 3 RESULTS

### 3.1 Body weight and visceral index

The results showed that there was no difference in the initial body weight of all groups. Meanwhile, the mice of MOD group showed a loss in final body weight compared to those in the NC group (*p* < .05; Table 2). However, the intervention of LN and HN groups (*p* < .05) increased the final body weight compared with the MOD group (Table 2). Data showed that NA treatment may affect the weight of mice. The organ index of liver and thymus in MOD group was decreased compared with the NC group (*p* < .01; Table 2). Meanwhile, the viscera coefficient of LN and HN groups (*p* < .05) were increased compared with the MOD group (*p* < .05). The results showed that NA effectively improved the organ index of mice induced by D‐galactose/AlCl_3_, and the improvement effect of each organ was different.

**TABLE 2:** Effect of NA on body weight and organ coefficient.

Group	Body weight (g)		Viscera index (mg/g)	
	Initial	Final	Liver	Thymus
NC	21.81 ± 1.10	27.68 ± 1.62	47.69 ± 2.64	1.28 ± 0.29
MOD	21.62 ± 1.23	25.37 ± 1.42^#^	44.11 ± 2.16^##^	0.81 ± 0.21^##^
LN	22.28 ± 0.92	27.53 ± 1.54*	47.04 ± 2.26*	1.08 ± 0.12*
HN	21.94 ± 1.04	27.58 ± 1.95*	47.18 ± 2.45*	1.23 ± 0.20**

*Note*: The results are presented as mean ± SD, *n* = 6. ^#^ *p* < .05 and ^##^ *p* < .01 versus the NC group; *p < .05 and **p < .01 versus the MOD group.

### 3.2 Behavioral experiments

#### 3.2.1 Open‐field test (OFT)

**FIGURE 1:**
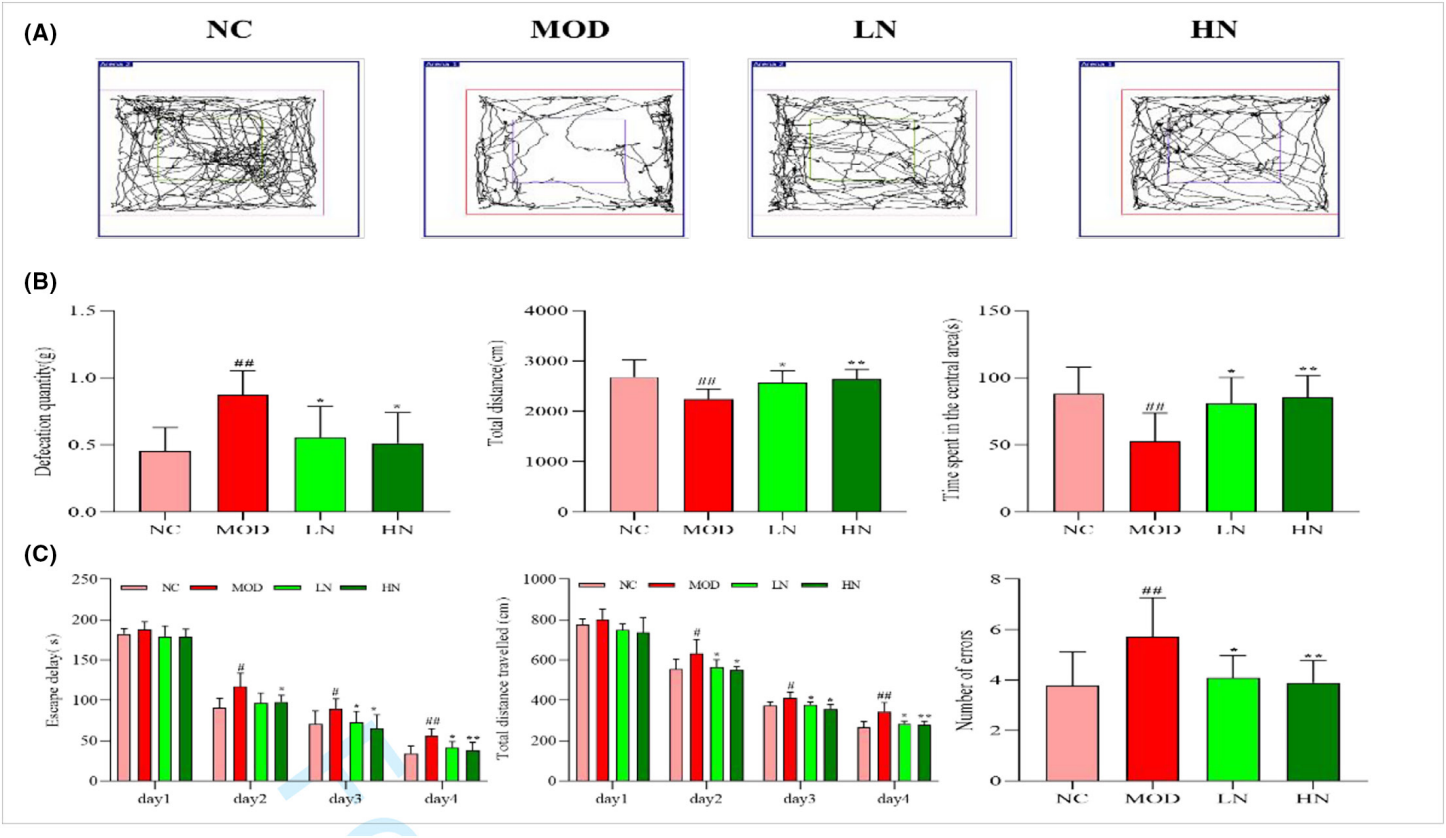
Learning and memory protective effects of NA in D‐galactose/AlCl_3_ induced mice. (a) Trajectory map of mice in the OFT. (b) Effect of NA on the defecation quantity, total distance, and time spent in the central area of the OFT. (c) Effect of NA on the escape delay, total distance traveled, and number of errors of the BM. The results are presented as mean ± SD, (*n* = 12). ^#^ *p* < .05 and ^##^ *p* < .01 versus the NC group; *p < .05 and **p < .01 versus the MOD group.

Figure 1a exhibits representative movement tracks of mice in each group. As shown in Figure 1b, the mice induced with D‐galactose/AlCl_3_ had a higher defecation quantity than the normal mice (*p* < .01) compared with the MOD group; however, mice treated with NA decreased the defecation quantity (*p* < .05). In addition, compared with the NC group, the total distance and the time spent in the central area were decreased in the mice of MOD group (*p* < .01). However, the intervention of NA reversed this trend. The HN group (*p* < .01) increased significantly compared with the LN group (*p* < .05). The results suggested that NA might have a positive effect in relieving anxiety and enhancing autonomous motor ability.

#### 3.2.2 Barnes maze test (BM)

The results showed that these indexes of MOD group are increased compared to NC group, including escape delay, distance traveled, and number of errors (*p* < 0.05, *p* < .01, and *p* < .01; Figure 1c). Meanwhile, these indexes decreased after NA intervention. The HN group (*p* < .01) decreased significantly compared with the LN group (*p* < .05). The results suggested that NA intervention could enhance the learning and memory abilities of mice (Figure 1c).

### 3.3 The analysis of antioxidant capacity and liver function index

**FIGURE 2:**
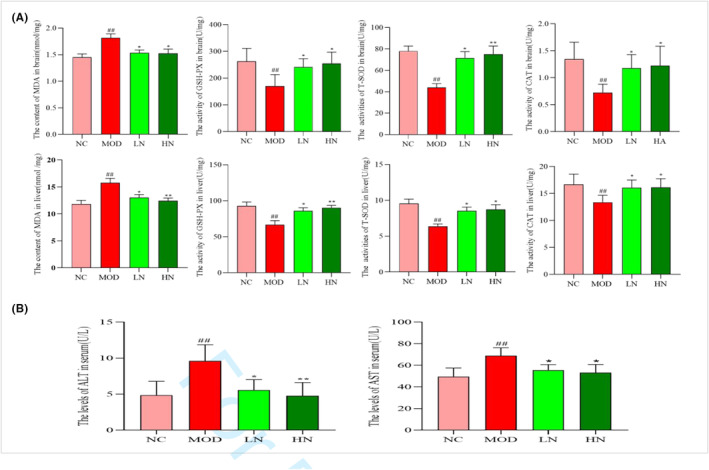
Effect of NA on related antioxidant indices in D‐galactose/AlCl_3_‐induced mice. (a) The activity of T‐SOD, GSH‐PX, CAT, and the content of MDA (b) The levels of ALT and AST. The results are presented as mean ± SD, *n* = 6. ^#^ *p* < .05 and ^##^ *p* < .01 versus the NC group; *p < 005 and **p < .01 versus the MOD group.

In this study, the activity of T‐SOD, GSH‐PX, and CAT was decreased after D‐galactose/AlCl_3_ intervention (*p* < .01). Meanwhile, the content of MDA in MOD group was significantly higher than that in NC group. However, compared with MOD group, these activities increased and MDA content decreased after NA treatment (*p* < .05; Figure 2a).

In addition, compared with the NC group, the levels of ALT and AST were increased in MOD group (*p* < .05; Figure 2b). Nevertheless, the levels of ALT and AST decreased after NA intervention. These results indicate that NA might have a positive effect on enhancing antioxidant capacity and protecting the liver.

### 3.4 The analysis of neurotransmitters and inflammation

**FIGURE 3:**
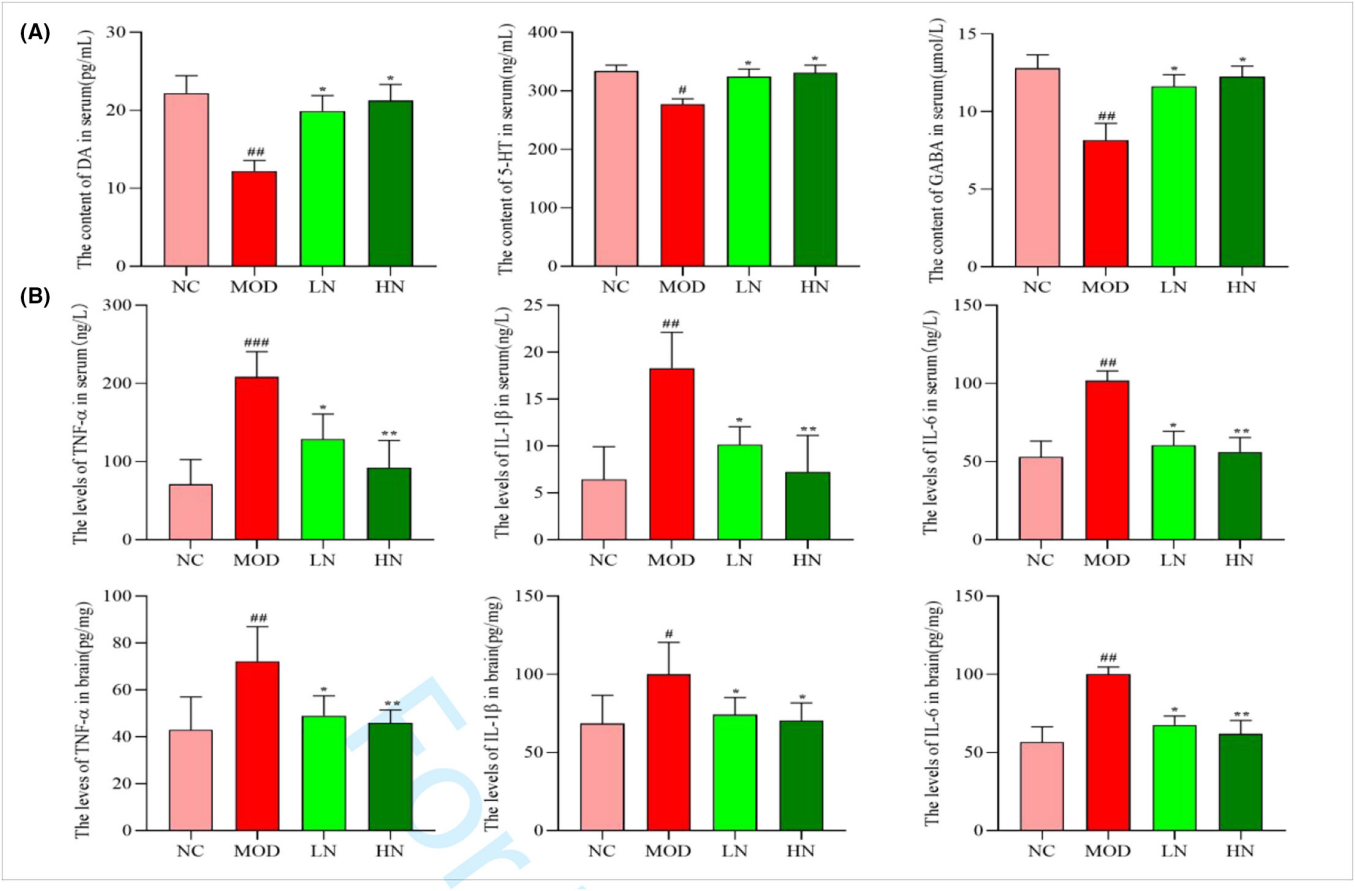
Effect of NA on the levels of neurotransmitters and inflammation in D‐galactose/AlCl_3_‐induced mice. (a) The content of 5‐HT, DA, and GABA in the serum. (b) The levels of TNF‐α, IL‐6, and IL‐1β in the serum and brain. The data are presented as mean ± SD (*n* = 6). ^#^ *p* < .05, ^##^ *p* < .01 and ^###^ *p* < .001 versus the NC group; *p < .05 and **p < .01 versus the MOD group.

The increased levels of DA, 5‐HT, and GABA are beneficial to alleviate AD. In this study, D‐galactose/AlCl_3_ treatment significantly reduced the levels of DA, GABA, and 5‐HT in serum compared to the NC group (*p* < .01, *p* < .05, and *p* < .01) (Figure 3a). However, compared with the MOD group, the content of DA, GABA, and 5‐HT was significantly increased after NA intervention (*p* < .05) (Figure 3a). In previous reports, the combined intervention of D‐galactose/AlCl_3_ increased the level of proinflammatory factors and led to the occurrence of tissue inflammation (Zhang et al., 2020). In this study, the levels of TNF‐α, IL‐1β, and IL‐6 in serum and brain were higher in the MOD group than that of NC group (*p* < .001, *p* < .01, and *p* < .01; Figure 3b). However, compared with the MOD group, NA treatment decreased the levels of TNF‐α, IL‐6, and IL‐1β (*p* < .01).

### 3.5 Hematoxylin–eosin staining (HE staining)

**FIGURE 4:**
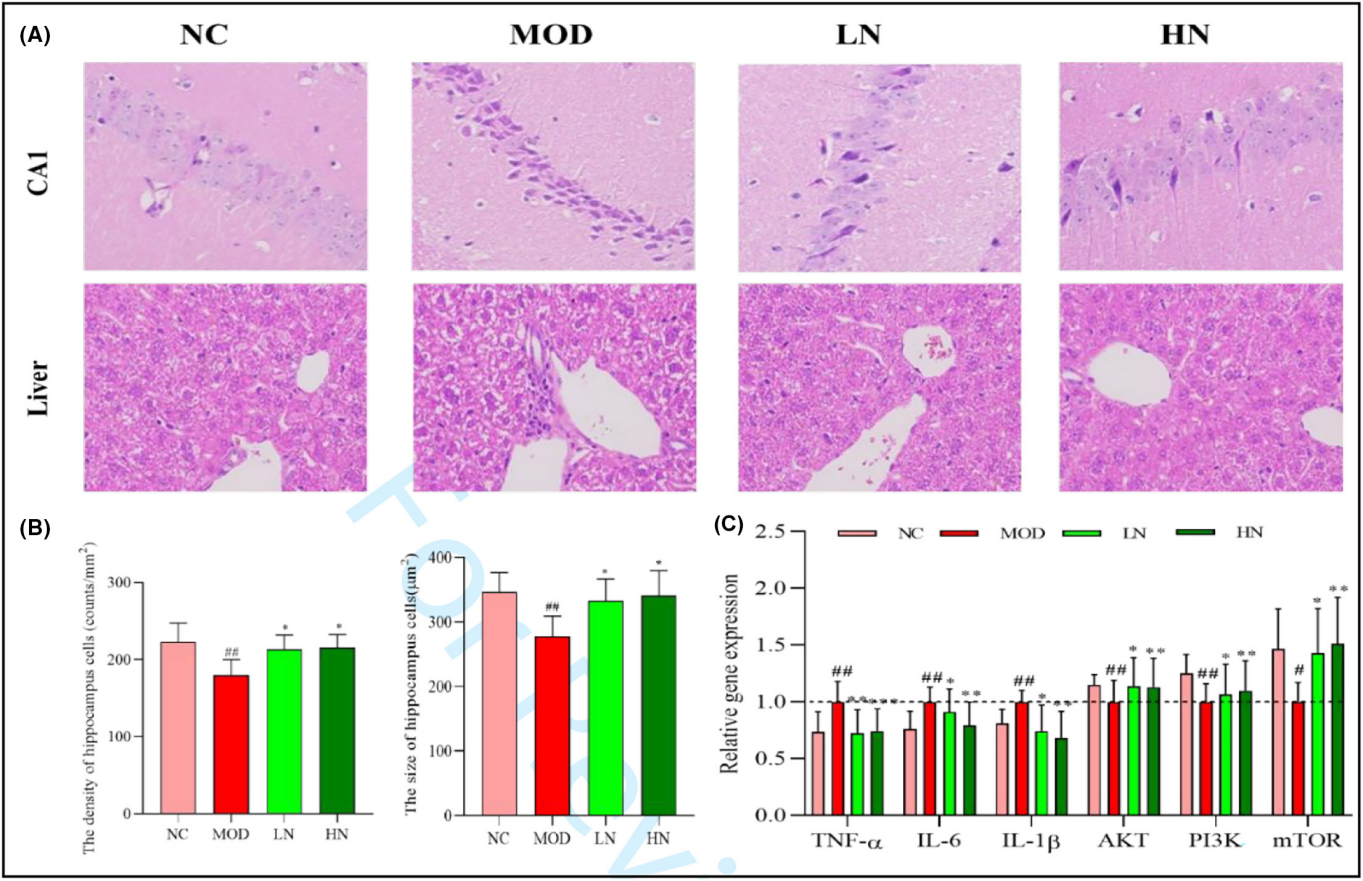
Effect of NA on the cell morphology and gene expression in D‐galactose/AlCl_3_‐induced mice. (a) Effect of NA on cell morphology in the CA1 (HE staining, magnification 400×) and liver tissue (HE staining, magnification 400×). (b) Effect of NA on cell density and size of hippocampus. (c) Effect of NA on gene expression of PI3K, AKT, mTOR, TNF‐α, IL‐6, and IL‐1β in the brain. The data are presented as mean ± SD (*n* = 6). ^#^ *p* < .05 and ^##^ *p* < .01 versus the NC group; *p < .05, **p < .01 and ***p < .001 versus the MOD group.

To observe whether NA acts as a protective agent in D‐galactose/AlCl_3_‐induced mice, HE staining of the hippocampus and liver of the mice is shown in Figure 4a. The hippocampal neurons in the cornu ammonis region 1 (CA1) were well preserved with a clear cell structure. By contrast, it was found that the phenomenon of irregular and apoptotic cells, as well as the nucleus pyknotic and deeply stained was increased in the MOD group. Nevertheless, the morphological damage was relieved with NA treatment (Figure 4a). Compared with NC group, the density and size of hippocampal cells in MOD group were decreased (*p* < .01). However, this trend was reversed after the intervention of NA (*p* < .05; Figure 4b).

The results of liver histopathology showed that the MOD group had more serious inflammation and invasion than the NC group, and the proportion of irregular cells and apoptotic cells increased significantly (Figure 4a). However, the intervention of NA relieved the morphological damage in the liver tissue induced by D‐galactose/AlCl_3_.

### 3.6 The gene expression of PI3K, AKT, mTOR, TNF‐α, IL‐6, and IL‐1β

At the genetic level, compared with the NC group, the gene expression levels of PI3K, Akt, and mTOR were significantly reduced in MOD group (*p* < .05) (Figure 4c). The expression levels of TNF‐α, IL‐6, and IL‐1β were significantly upregulated by treatment with D‐galactose/AlCl_3_ (*p* < .01). However, NA intervention upregulated the expression levels of PI3K, AKT, and mTOR (*p* < .01, *p* < .05), and downregulated the expression levels of TNF‐α, IL‐6, and IL‐1β (*p* < .001, *p* < .01; Figure 4c).

## 4 DISCUSSION

In the world, AD is considered to be the most common cause of dementia, and the number of people affected is increasing dramatically (Clark et al., 2021; Teleanu et al., 2022). Notably, the increase of oxidative stress and inflammation levels can accelerate the pathogenesis of AD (Clark et al., 2021; Hu, Cui, & Zhang, 2021; Hu, Wharton, & Parker, 2021; Xiong et al., 2020). Therefore, how to mitigate the adverse effects of oxidative stress and inflammation on the organism is considered as the main way to alleviate AD. It has been found that the combined intervention of D‐galactose and aluminum chloride undergoes metabolism in the body to produce D‐galactitol that is not metabolized by the organism (which causes an increase in osmotic pressure and disrupts the normal morphology of hippocampal neurons) and causes a progressive loss of neurological function (Dong & Liu, 2021; Oskouei et al., 2021; Wei et al., 2017). Furthermore, NA is ultra‐long‐chain monounsaturated fatty acid (C24:1) with antioxidant, immunity‐enhancing, and memory‐improving effects, and promotes the growth of neuronal cells, but the mechanism of action needs to be further explored (Liu, Sun, et al., 2021; Liu, Wang, et al., 2021; Nakata & Fujita, 2020).

In general, the onset of AD is often accompanied by atrophy of the brain, liver, and thymus (Batista et al., 2020). In this study, the organ index of liver and thymus of mice induced by D‐galactose/AlCl_3_ was apparently lower than the NC group (Table 1). However, NA intervention alleviated D‐galactose/AlCl_3_‐induced organ atrophy. This is similar to the previous report that the NA relieved the visceral atrophy induced by 1‐Methyl‐4‐phenyl‐1,2,3,6‐tetrahydropyridine (MPTP; Hu, Cui, & Zhang, 2021; Hu, Wharton, & Parker, 2021).

The levels of increased anxiety and decreased learning capacity are the main manifestations of AD. Dandong Hu et al found that the climbing time was dramatically decreased after the NA treated compared to AD mice in the pole test. At the same time, they also found that the NA‐treated mice showed more spontaneous movements in OFT (Hu, Cui, et al., 2021, Hu, Wharton, et al., 2021) compared to the PD mice, which is similar to our results. In this study, compared with the NC group, the total distance and the time spent in the central area were decreased in the MOD group (Figure 1b). However, NA treatment reversed this situation. In addition, the results of BM show that the mice of NA group need less time than MOD group to find the escape box (Figure 1c). These results imply that NA has a positive effect in alleviating anxiety and improving memory of AD mice.

D‐galactose/AlCl_3_ can cause a series of degenerative changes in cells, including decreased number of nerve cells and abnormal cell morphology (Wu et al., 2021). Meanwhile, studies have found that NA intervention can significantly alleviate morphological abnormalities of hippocampus caused by oxidative stress, such as the decreased proportion of pyknosis and apoptosis cells (Vozella et al., 2017). In addition, the theory of the liver–brain axis says that chronic inflammatory liver diseases are often accompanied by behavior changes, including fatigue and cognitive dysfunction (D'Mello & Swain, 2014). This is similar to our experimental results. In this study, irregular cells and inflammatory infiltration in liver and hippocampus were serious in mice treated with D‐galactose/AlCl_3_. However, these phenomena were relatively alleviated after NA intervention (Figures 3 and 4). This indicated that NA had a protective effect on cell morphology. Liver damage caused by oxidative stress is often accompanied by an increase of ALT and AST levels (Qiu et al., 2021). In addition, it was found that ALT and AST levels of serum were decreased in C57BL/6 mice induced by MPTP after the intervention of NA (60 mg/kg) (Hu, Cui, et al., 2021, Hu, Wharton, et al., 2021). In this study, NA intervention reduced ALT and AST levels in D‐galactose/AlCl_3_‐induced AD mice (Figure 2). This suggests that NA may have a protective effect on liver function against the damage caused by oxidative stress.

Excessive production of ROS can destroy the normal structure of nerve cells, attack the oxidative phosphorylation system of the body, and cause AD. In previous reports, it was found that NA intervention can reduce the adverse effects of 6‐hydroxydopamine on PC12 cells, including enhancing the activity of SOD and reducing the production of MDA (Umemoto et al., 2021). Meanwhile, NA intervention enhanced GSH‐PX activity in hippocampus and increased neurotransmitter level in normal Kunming mice (Wu et al., 2021). In this study, NA enhanced the activity of T‐SOD, GSH‐PX, and CAT, and decreased the content of MDA in liver and brain (Figure 2a). This further confirms that NA has antioxidant effects in vivo.

The decline of neurotransmitters level is correlative with brain dysfunction. In previous reports, excessive aluminum ions can cause neurodegeneration and decrease the level of neurotransmitters, such as dopamine, serotonin, and GABA (Song et al., 2022). Meanwhile, D‐galactose can accelerate this process by inducing ROS production (Ameen et al., 2022; Zhao et al., 2021). However, NA intervention attenuated the decline in neurotransmitter levels caused by D‐galactose/AlCl_3_ in this study (Figure 3a). In addition, some research found that the pro‐inflammatory elements (such as TNF‐α and IL‐1β), when expressed, affect the neuronal processes in AD brain (Khalid et al., 2020). In this study, we found that NA decreased the levels of TNF‐α, IL‐1β, and IL‐6 (Figure 3b), suggesting that NA has a positive effect in reducing neuroinflammation.

The activation of PI3K pathway contributes to relieving cognitive impairment caused by oxidative stress (Li et al., 2021). In previous study, the escape delay of traumatic brain injury rats in water maze test was shortened, and the survival rate of hippocampal neurons was significantly increased after PI3K pathway was activated (Wang et al., 2017). In this study, the gene expression levels of PI3K, AKT, and mTOR were upregulated. In addition, the gene expression levels of TNF‐α, IL‐1β, and IL‐6 were downregulated after NA intervention (Figure 4c). These evidences suggest that NA may exert potent protective effect by activating PI3K pathway.

## 5 CONCLUSION

In general, NA alleviated the organ atrophy and anxiety caused by AD and improved the cognitive and learning abilities of D‐galactose/AlCl_3_‐induced mice. Meanwhile, it enhanced the antioxidant capacity in vivo, reduced the level of proinflammatory factors, and maintained the normal morphology of cells. In addition, NA intervention upregulated the expression of PI3K pathway genes. However, some issues will require further study in the future: first, because bioavailability is a crucial factor for neuroprotective agent; therefore, the absorption and transportation of NA need to be studied in the targeted area; Second, there are different research results on whether NA can pass the blood–brain barrier, this needs further study. In a word, although there are some limitations in the current study, due to its antioxidant and neuroprotective abilities, nervonic acid still has the potential to intervene in AD.

## AUTHOR CONTRIBUTIONS

**Mayile Aihaiti:** Conceptualization (equal); writing – original draft (equal); writing – review and editing (equal). **Haidan Shi:** Writing – review and editing (supporting). **Yaojie Liu:** Writing – review and editing (supporting). **Chen Hou:** Methodology (supporting). **Xiaoyu Song:** Investigation (supporting). **Mengting Li:** Writing – review and editing (supporting). **Jianke Li:** Conceptualization (equal); formal analysis (equal); funding acquisition (equal); project administration (equal).

## CONFLICT OF INTEREST STATEMENT

The authors declare no conflict of interest in this study.

## Data Availability

The data that support the findings of this study are available on request from the corresponding author.
